# Infants Help a Non-Human Agent

**DOI:** 10.1371/journal.pone.0075130

**Published:** 2013-09-18

**Authors:** Ben Kenward, Gustaf Gredebäck

**Affiliations:** Department of Psychology, Uppsala University, Uppsala, Sweden; Goldsmiths, University of London, United Kingdom

## Abstract

Young children can be motivated to help adults by sympathetic concern based upon empathy, but the underlying mechanisms are unknown. One account of empathy-based sympathetic helping in adults states that it arises due to direct-matching mirror-system mechanisms which allow the observer to vicariously experience the situation of the individual in need of help. This mechanism could not account for helping of a geometric-shape agent lacking human-isomorphic body-parts. Here 17-month-olds observed a ball-shaped non-human agent trying to reach a goal but failing because it was blocked by a barrier. Infants helped the agent by lifting it over the barrier. They performed this action less frequently in a control condition in which the barrier could not be construed as blocking the agent. Direct matching is therefore not required for motivating helping in infants, indicating that at least some of our early helpful tendencies do not depend on human-specific mechanisms. Empathy-based mechanisms that do not require direct-matching provide one plausible basis for the observed helping. A second possibility is that rather than being based on empathy, the observed helping occurred as a result of a goal-contagion process in which the infants were primed with the unfulfilled goal.

## Introduction

From early in their second year children help adults unable to reach their goals [1]–[4]. Motivations for prosocial behaviour in young children are diverse [5]–[7], but one important motivation for helping in young children is an intrinsic sympathy-based feeling of altruism towards the individual in need of help [8]–[10]. This is demonstrated by studies showing that from 18 months children are more likely to help victims of anti-social acts [11], [12] and that helping is inhibited rather than promoted by rewards in 20-month-olds [13]. Furthermore, 24-months-old’s physiological arousal produced in response to an individual in need is reduced not only when the children provide the necessary help, but also when help is provided by a third party, indicating that help is motivated by a basic sympathetic concern for the individual’s welfare [14].

Although motivations for young children’s helping are therefore beginning to be understood, the underlying neural mechanisms remain unclear. To explain empathy in adults, and thus sympathy and helping, one prominent type of mechanistic account invokes the mirror system. (Empathy refers to the sharing of an emotion with another, whereas sympathy refers to the feeling of concern for another’s wellbeing which can be evoked by empathy, and which can motivate helping [15]). The mirror system is highly complex and includes numerous different pathways which might support empathy [16]–[20], but here we focus on just one frequently highlighted type of mirror activity known as direct matching.

We use the term direct-matching as originally defined, as a “mechanism that directly maps a pictorial or kinematic description of the observed action onto an internal motor representation of the same action” [21] (see also [22], [23]). In other words, an observed action is represented by an action plan for performing the same action. It is argued that “the precise kinesthetic aspects of the movement (for example, how much the finger should be lifted)” are encoded [21].

Direct matching is known to play a causal role in social understanding [24]. One specific way in which it might enable empathy is by enabling observers’ perceptions of others’ facial expressions to be directly linked to the experience of displaying the same expression [25]–[27]. A more general mechanism by which direct matching might lead to helping is that direct matching assists the observer to empathically and vicariously experience an observed challenging situation, with the experience of empathy leading to sympathy and thus a desire to help [9], [28], [29]. Molnar-Szakacs explains the link between direct matching and empathy thus: “Empathic emotional attunement appears to rely on the direct link between perception and action instantiated by the human MNS [mirror neuron system]. As perceiving an action activates the same representations as performance of the same action, this overlap might allow humans to ‘embody’ the behavior of others and to infer their internal states, including the intentions and emotions driving [them]” [29]. According to the direct matching account of empathic helping, therefore, direct matching enables empathy, which leads to sympathy and a consequent desire to help.

Given the above arguments, we postulate that it is plausible that direct-matching is a prerequisite for helping in infants, because it is not certain that any other neural mechanisms for empathic and non-empathic helping are operational in infancy. The purpose of the current study is to test this strong hypothesis. It makes the strong prediction that infants would not help a geometric-shape agent lacking human-isomorphic body-parts because such an agent cannot elicit direct matching which by definition requires at least some degree of isomorphism of movable body parts [22]. This prediction has not to our knowledge been tested, but it is not implausible that infants might help such an agent. The extraction of social meaning from the movements of geometric-shape agents begins in early infancy [30]–[32]. Infants evaluate such agents’ helpful acts as positive and hindering acts as negative [33]–[35] (but see [36]), with even three-month-olds possessing the rudiments of this ability [37]. These results indicate that mechanisms independent of direct-matching are important for infants’ social cognition. However, as the mirror system is also active in infants [38], and as active helping may not be based on the same systems as evaluation of others’ helping, it remains unclear what underlying neural mechanisms motivate infants’ own acts of helping. Furthermore, although empathy is clearly an important motivator for helping in young children, it is also possible that mechanisms not based on empathy might play a role. It may be that a goal-contagion priming account [39] might explain some aspects of infant helping. According to this account, which is addressed further in the discussion, the encoding of an agent’s goal leads to the adoption of the same goal in a priming process akin to automatic imitation.

Here, in the experimental condition, a geometric-shape agent’s apparent goal is on the other side of a barrier. On reaching the barrier the agent first travels up and down the length of it and then repeatedly knocks into it as if attempting to force a way through. Infants can help the agent by lifting it over the barrier. Only accounts of helping not requiring direct matching predict that infants will do so. The numerous explanations for why infants might lift the agent over without intending to help it, such as exploratory behaviour, are controlled for in a condition in which everything is identical except that the barrier is incomplete. In this condition the agent’s identical action of travelling up and down is instead intended to indicate that there is a clear passage to the other side which the agent chooses not to take. Unlike in the experimental condition, there is therefore no obvious intended unsuccessful action. As infants are therefore much less likely to perceive an unfulfilled goal, hypotheses of helping do not predict that infants will lift the agent beyond the barrier, because this would not fulfil an incomplete action. In summary, if mechanisms motivating helping without direct matching exist, infants are predicted to lift the agent over the barrier when the barrier is complete, but to do so less frequently when the barrier is incomplete. Because the hypothesis of imitative goal contagion predicts that infants re-enact the agent’s original actions (knocking the barrier rather than moving over it) we also examine this.

## Materials and Methods

### Ethics Statement

The work conducted in this study was given written approval by the Uppsala Regional Ethics Committee (Regionala etikprövningsnämnden i Uppsala, application reference number 2009/103). Infants’ parents gave informed written consent.

### Participants

Sixty 17-month-olds (27 girls; mean age 17.5, SD = .7) were randomly divided between the experimental or control conditions. An additional 7 infants were excluded from analysis because of parental interference (1), technical problems (2), or because of fussiness before a minimum criteria of three trials were reached (5). Prior to the experiment, all parents were informed that we were interested in whether infants would help, but not that there were two conditions.

### Procedure

**Figure 1:**
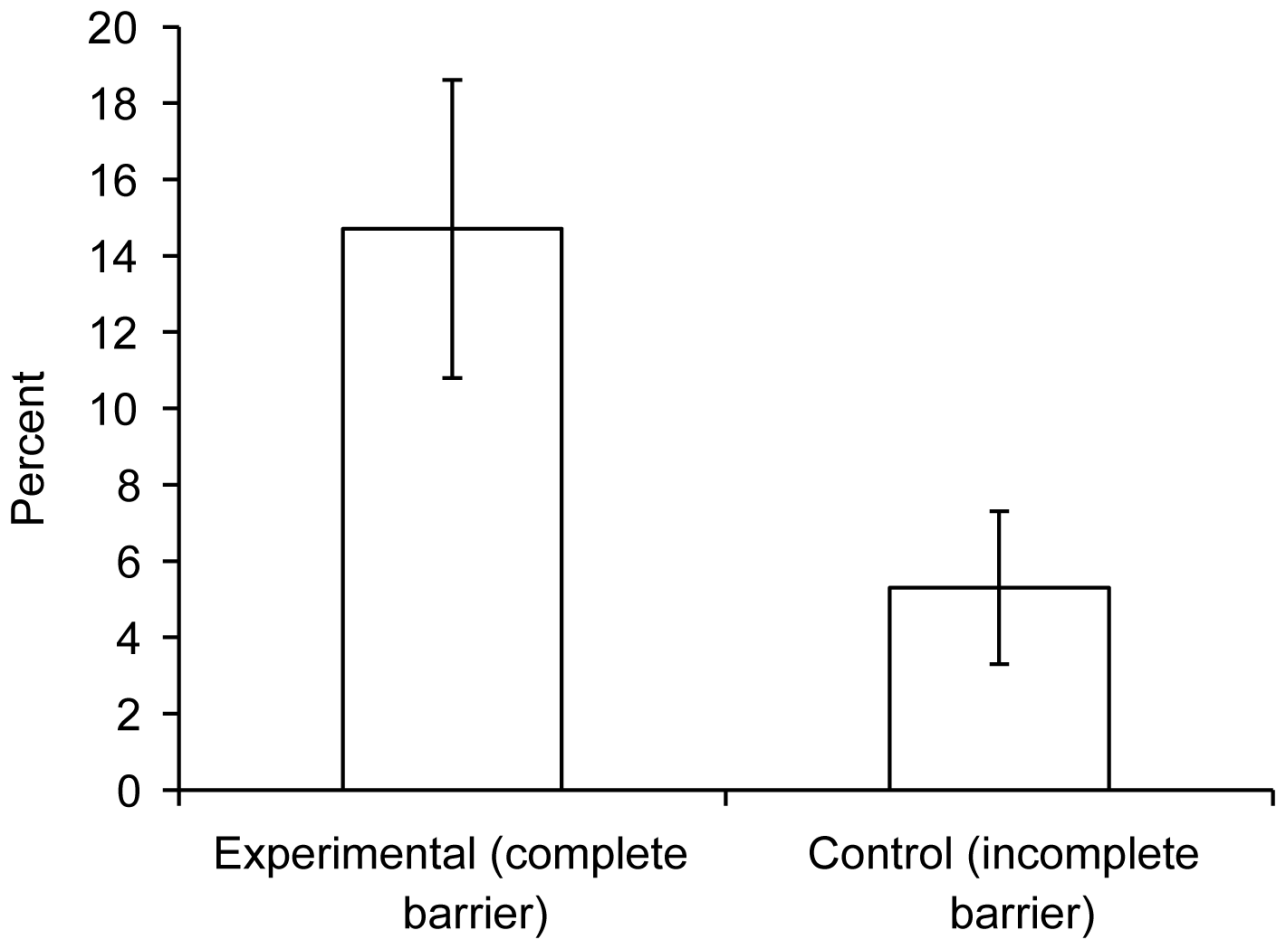
Infants’ view at the start of the trial. (A) Experimental condition. (B) Control condition.

Each infant participated until it became fussy or until six trials were completed. Each trial was identical and began with the infant sitting in the parent’s lap just out of reach of the table. Parents held their infants around the waist only. The table was divided into two by a barrier composed of three wooden blocks in the experimental condition, but in the control condition only the central wooden block was present (Figure 1). This was the only difference between conditions. A screen attached to the back of the table hid the experimenter as she sat behind moving the agent using a magnet under the table. The agent, a slightly elongated yellow ball with fabric eyes and small enough for infants to lift, was initially positioned to the left of the barrier. On the right side was a larger pink ball with fabric eyes, positioned on a pink shape, besides which was an unoccupied yellow shape intended to enhance the impression of an intended goal for the yellow agent. The gap between the blocks was one third of the diameter of the agent – from three months infants do not expect objects to pass through gaps smaller than themselves [40].

Trials began with the agent travelling towards the central block, and on reaching its left side, travelling up and down the table. With identical movements, therefore, in the experimental condition the agent travelled up and down the length of the barrier, whereas in the control condition the agent moved past the empty spaces to the sides of the central block. After this the agent began to knock, hard and at speed, into the central block, with each knock followed by a slower backwards retreat (Videos S1 and S2 show the experimental and control conditions respectively). Each knock came from a slightly different angle, serving to reinforce the impression of agency rather than mechanical movement [41]. Parents were instructed to move forward after five knocks so that their infant could reach the agent. After this point, knocking continued until the infant began moving the agent or until 15 seconds had passed, at which latter point the trial was terminated. Once the infant had begun moving the agent, the trial was terminated either when the infant ceased contacting the agent or after an additional 15 seconds. After the trial, the experimenter retrieved the agent, the parent rotated the chair so the infant could not see the table, and the experimenter replaced the agent in the starting position.

### Stimulus Validity

To confirm that adults at least readily interpreted the agent in the experimental condition as an agent attempting to cross the barrier and in need of help, but made this interpretation less readily in the control condition, a convenience sample of 15 hypothesis-blind non-psychologist adults (mean age 44 years, *SD* = 11, 7 women) was recruited and tested via the internet. Participants were displayed movies of both conditions in counterbalanced order (Videos S1 and S2), and after each movie were asked “what is your immediate intuitive interpretation of what you just saw?” and “if you could intervene in this situation, what would you do?” One subject was excluded for stating only that the movies were “silly”. All 14 adults described the agent as an agent in both conditions. The agent was marginally more likely to be described as attempting to travel past the barrier in the experimental condition (100%) than in the control condition (64%), *p* = .074, McNemar’s test. Adults were more likely to state they would help the agent past the barrier in the experimental condition (100%) than in the control condition (57%), *p* = .041, McNemar’s test. Adults were marginally more likely to state that the agent’s goal was to knock the barrier in the control condition (36%) than in the experimental condition (0%), *p* = .062, McNemar’s test.

### Coding and Analysis

Two coders, one of whom was blind to the study hypothesis, coded each trial from video. The following behaviours were coded: whether the infant moved the agent beyond the barrier (defined as leaving the agent on the table to the right of the right-hand edge of the central block; for inter-observer agreement Cohen’s κ = .95); whether the infant placed the agent on the yellow shape beyond the barrier (κ = .95); whether the infant moved the agent at all (κ = .91); and whether the infant replicated the agent’s original actions (defined as knocking it into the barrier or sliding it rhythmically back and forth on the table; κ = .64). The blind coding was used for analysis.

Two-sample *t*-tests were conducted using Welch’s standard correction for possible non-homogeneity of variance. Because data was in the form of proportions and left-skewed due to many zero values, non-parametric two-sample permutation tests were also conducted. The standard method was used of comparing the *t*-statistic with a null-hypothesis distribution generated by randomly permuting the data, rather than with the parametric null-hypothesis distribution [42]. To generate the permutation null-hypothesis distribution, the standard method was used: the two samples were pooled and then divided into two randomly selected samples one million times, with the randomised *t*-statistic calculated each time.

## Results

**Figure 2:**
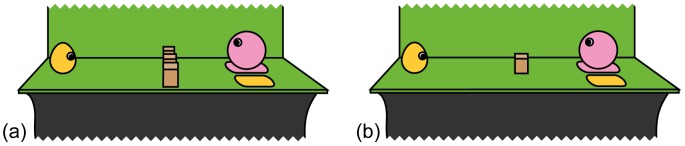
Mean percentage of trials participant moves agent beyond barrier, by condition. Error bars show one standard error.

Moving the agent beyond the barrier occurred on a higher proportion of trials in the experimental condition (Table 1, Figure 2). Video S3 shows an infant in the experimental condition lifting the agent over the barrier. The same result was obtained when the number of trials each infant lifted the agent over the barrier was expressed as a proportion of trials in which the infant moved the agent, instead of as a proportion of trials the infant completed (Table 1). The agent was moved beyond the barrier at least once by 40% of participants in the experimental condition and 23% of participants in the control condition. No significant difference was detected in the proportion of trials the agent was lifted beyond the barrier in which the agent was placed on the yellow shape, although sample sizes were small due to the low frequencies of lifting over the barrier (Table 1). Re-enactment of the agent’s original actions was very infrequent in both conditions (Table 1).

**Table 1:** Proportions of trials containing specific behaviours.

	Experiment			Control							
	*M*	*SD*	*n*	*M*	*SD*	*n*	*t*	*d.f.*	*p_t_*_-test_	*p_permutation_*	*d*
Proportion of trials in which the infant movedthe agent beyond the barrier	.15	.21	30	.05	.11	30	2.14	42	**.039**	**.039**	.55
Proportion of trials in which the infant movedthe agent in which the agent wasmoved beyond the barrier	.21	.29	27	.07	.13	26	2.18	51	**.034**	**.032**	.60
Proportion of trials in whichthe infant moved the agent beyond the barrier in which the infantplaced the agent on the yellow square	.44	.54	12	.21	.39	7	1.16	14	.264	.289	.54
Proportion of trials completed before fussiness	.97	.08	30	.96	.08	30	0.26	58	.795	1.000	.07
Proportion of trials in which theinfant moved the agent	.72	.33	30	.59	.37	30	1.47	57	.148	.149	.38
Proportion of trials in which the infantre-enacted the agent’s original actions	.06	.14	30	.06	.18	30	0.03	55	.979	.949	.01

There was no evidence that the conditions differed in how they engaged the participants’ attention and activity. The mean proportion of trials completed before fussiness was the same for both conditions, and no difference was detected in the proportion of completed trials in which the infant moved the agent (Table 1).

## Discussion

Although moving the agent beyond the barrier was infrequent compared to moving the agent in other ways, it did occur, and importantly, it occurred much more frequently in the experimental condition than in the control condition. Although there are many other reasons apart from helping (such as exploration) for why infants might move the agent beyond the barrier, these reasons generally apply equally to the control condition. It is not impossible to conceive of non-help-based hypotheses which might explain the condition difference – for example it may be inherently more rewarding to lift a ball past three blocks than past one – but such post-hoc hypotheses lack the plausibility conferred on the helping hypothesis by previous results concerning infants’ helpful tendencies and interpretations of non-human goal-directed action. Note also that our analysis of general exploratory behaviour did not indicate differences between the conditions. We therefore conclude that at least some of the observed transportations over the barrier were motivated by a tendency to help, by which we mean a tendency to act in a way facilitating the achievement of another individual’s goal.

Based on the conclusion that infants helped a non-human agent, the following further conclusions about the underlying mechanisms of helping can be made. The results cannot be explained by the direct-matching mirror account of empathy-based helping, and direct-matching mirror mechanisms are therefore not the only and perhaps not even the primary mechanisms for motivating help in infants. This does not, however, imply that direct-matching mirror mechanisms do not play a role when infants help human agents. It should be noted that in comparison to rates of helping of adult strangers by 18-month-olds [43], the rates of helping observed here were very low. While we have established that direct-matching is not a prerequisite for helping in infants, the finding that helping rates are comparatively low for a non-human agent suggests that human-specific mechanisms such as direct matching are likely to play an important role in motivating helping of humans. Such human-specific mechanisms might relate to action understanding, empathy, and/or sympathy. It should also be noted that instrumental helping is found at 14 months [1], and helpful communication at 12 months [4], so our findings in 17-month-olds do not necessarily speak to the very earliest forms of helping. As the relatively low rates of helping here imply that human specific mechanisms are likely to be important at 17 months, it is plausible that helping before 17 months does rely on human specific mechanisms.

Our findings further reinforce the point that direct matching is not a prerequisite for understanding others’ actions in infants and adults [44]–[47]. It is also relevant to note that although the issue is still a subject of much debate [23], recent studies have questioned the extent to which direct matching occurs and the extent to which it plays a causal role in action understanding [48]–[50]. For example, nine-month-olds have been demonstrated to show motor activation when observing actions they could not themselves perform, suggesting that some forms of matching may be the consequence of planning an action similar but not identical to the one observed, rather than the cause of understanding the action [51]. Motor activation, including forms of mirror activity which are not direct-matching, is therefore not ruled out here as being involved in participants’ interpretations of the agent’s actions.

What mechanisms did therefore account for the observed helping? Some prior observations allow informed speculation. Many neural mechanisms involved in empathy in adults and older children do not involve direct matching [17], [18], [52], and it is likely that these may play a role in infancy. More specifically, aspects of empathy depend on connections between emotion centres (particularly the amygdala, the insula, and the anterior cingulate cortex) and the prefrontal cortex, both in older children [53] and in adults [54]–[56]. We note that the amygdala also plays a key causal role in allowing the actions of animated geometric-shapes to be evaluated in terms of social meaning (known as anthropomorphizing [57]). We suggest therefore that a plausible account of empathy for and thus helping of geometric-shape agents is based upon a network with the amygdala at its centre, because the amygdala plays a key role both in perceiving such agents’ movements as actions with social meaning, and in assigning emotional valence to these actions.

A second possible explanation for the observed helping is a non-sympathy-based priming mechanism. The representation of the observed goal may have primed behaviour resulting in that goal, in a similar process to the goal contagion which has been observed in adults [39]. Note that in this case, helping can be seen as a similar process to automatic imitation [58]. Observation of non-human action primes motor activity in nine-month-olds [51], priming can increase helping frequency in 18-month-olds [59], and 18-month-olds are known to be able to imitate complete actions even when demonstrated incompletely [60]. Related to this, it has been argued that when young children observe unmet needs, they can sometimes be motivated to help not because of sympathy but because of a broader motivation to cause goals to be reached which is not predicated on an understanding of the self-other distinction [61]. One result, however, speaks against the goal-priming account. If goal-priming led to imitation of a non-human agent’s actions by infants, re-enactment of the agent’s original actions would be expected, at least in the control condition where there was no obvious incomplete action. Such re-enactment was observed only at very low frequencies, suggesting that goal-priming may not have been a strong motivator of the infants’ actions.

An issue concerning the validity of the method must be raised. Parents in both conditions were informed that we were investigating if infants would help the agent. Parents were not aware of condition differences, and were asked not to influence their infants’ behaviour. However, as adults also report that they would be more likely to lift the agent over the barrier in the experimental condition, some parents might have attempted to cause their infants to do so. Parents held their infants around the waist only and advanced and retreated from the table only at predetermined points. It would therefore have been challenging for them to influence the details of their infants’ manipulation of the agent. More plausibly parents might have been able to influence whether their infants picked up the agent, but no condition difference was detected, indicating that parental influence was unlikely to differ between conditions. Further, any undetected difference in influence on picking up could not have entirely accounted for differences in lifting over the barrier because it was more frequent in the experimental condition even when expressed as a proportion of trials in which the agent was picked up. Although we therefore argue that parental influence is an unlikely explanation for our result, future designs should remove this possibility entirely, for example by blindfolding parents.

In conclusion, the finding that non-direct-matching-based mechanisms can result in helping in infants gives further support to the idea that they play a prominent role more generally in human helping behaviour [17], [18], [52]. What this study most clearly demonstrates is that by late infancy humans’ helpful tendencies are built not only upon direct-matching mirror mechanisms in which others are perceived as “like me” [62], but also on more general mechanisms which can process non-human agents and their unachieved goals.

## Supporting Information

Video S1 **Infants’ view of the experimental condition.**
(MOV)

Video S2 **Infants’ view of the control condition.**
(MOV)

Video S3 **An infant in the experimental condition lifting the agent over the barrier.** The parent of the participant in this video has given written informed consent, as outlined in the PLOS consent form, to publish this video.
(MOV)

## References

[1] Warneken F, Tomasello M (2007) Helping and cooperation at 14 months of age. Infancy 11: 271–294. 10.1111/j.1532-7078.2007.tb00227.x 33412734

[2] Svetlova M, Nichols SR, Brownell CA (2010) Toddlers’ prosocial behavior: From instrumental to empathic to altruistic helping. Child Development 81: 1814–1827. 21077866 10.1111/j.1467-8624.2010.01512.x PMC3088085

[3] Rheingold HL (1982) Little children’s participation in the work of adults, a nascent prosocial behavior. Child Development 53: 114–125.

[4] Liszkowski U, Carpenter M, Tomasello M (2008) Twelve-month-olds communicate helpfully and appropriately for knowledgeable and ignorant partners. Cognition 108: 732–739. 18721918 10.1016/j.cognition.2008.06.013

[5] Thompson RA, Newton EK (2013) Baby altruists? Examining the complexity of prosocial motivation in young children. Infancy 18: 120–133.

[6] Brownell CA (2013) Early development of prosocial behavior: Current perspectives. Infancy 18: 1–9. 25632273 10.1111/infa.12004 PMC4306462

[7] Dunfield K, Kuhlmeier VA, O’Connell L, Kelley E (2011) Examining the diversity of prosocial behavior: Helping, sharing, and comforting in infancy. Infancy 16: 227–247. 10.1111/j.1532-7078.2010.00041.x 32693496

[8] Warneken F, Tomasello M (2009) The roots of human altruism. British Journal of Psychology 100: 455–471. 19063815 10.1348/000712608X379061

[9] de Waal FBM (2008) Putting the altruism back into altruism: The evolution of empathy. Annual review of psychology 59: 279–300. 10.1146/annurev.psych.59.103006.093625 17550343

[10] Hepach R, Vaish A, Tomasello M (2013) A new look at children’s prosocial motivation. Infancy 18: 67–90.

[11] Vaish A, Carpenter M, Tomasello M (2009) Sympathy through affective perspective taking and its relation to prosocial behavior in toddlers. Developmental Psychology 45: 534–543. 19271837 10.1037/a0014322

[12] Eisenberg N, Miller PA (1987) The relation of empathy to pro-social and related behaviors. Psychological Bulletin 101: 91–119. 3562705

[13] Warneken F, Tomasello M (2008) Extrinsic rewards undermine altruistic tendencies in 20-month-olds. Developmental Psychology 44: 1785–1788. 18999339 10.1037/a0013860

[14] Hepach R, Vaish A, Tomasello M (2012) Young children are intrinsically motivated to see others helped. Psychological Science 23: 967–972. 22851443 10.1177/0956797612440571

[15] Decety J (2010) The neurodevelopment of empathy in humans. Developmental Neuroscience 32: 257–267. 20805682 10.1159/000317771 PMC3021497

[16] Baird AD, Scheffer IE, Wilson SJ (2011) Mirror neuron system involvement in empathy: A critical look at the evidence. Social Neuroscience 6: 327–335. 21229470 10.1080/17470919.2010.547085

[17] Shamay-Tsoory SG (2011) The neural bases for empathy. Neuroscientist 17: 18–24. 21071616 10.1177/1073858410379268

[18] Zaki J, Ochsner K (2012) The neuroscience of empathy: progress, pitfalls and promise. Nature Neuroscience 15: 675–680. 22504346 10.1038/nn.3085

[19] Gallese V (2003) The roots of empathy: The shared manifold hypothesis and the neural basis of intersubjectivity. Psychopathology 36: 171–180. 14504450 10.1159/000072786

[20] Cheng Y, Yang C-Y, Lin C-P, Lee P-L, Decety J (2008) The perception of pain in others suppresses somatosensory oscillations: A magnetoencephalography study. Neuroimage 40: 1833–1840. 18353686 10.1016/j.neuroimage.2008.01.064

[21] Iacoboni M, Woods RP, Brass M, Bekkering H, Mazziotta JC, et al (1999) Cortical mechanisms of human imitation. Science 286: 2526–2528. 10617472 10.1126/science.286.5449.2526

[22] Gallese V, Rochat M, Cossu G, Sinigaglia C (2009) Motor cognition and its role in the phylogeny and ontogeny of action understanding. Developmental Psychology 45: 103–113. 19209994 10.1037/a0014436

[23] Rizzolatti G, Sinigaglia C (2010) The functional role of the parieto-frontal mirror circuit: interpretations and misinterpretations. Nature Reviews Neuroscience 11: 264–274. 20216547 10.1038/nrn2805

[24] Elsner C, D’Ausilio A, Gredebäck G, Falck-Ytter T, Fadiga L (2013) The motor cortex is causally related to predictive eye movements during action observation. Neuropsychologia 51: 488–492. 23267825 10.1016/j.neuropsychologia.2012.12.007

[25] Iacoboni M (2009) Imitation, empathy, and mirror neurons. Annual review of psychology 60: 653–670. 10.1146/annurev.psych.60.110707.163604 18793090

[26] Bastiaansen JACJ, Thioux M, Keysers C (2009) Evidence for mirror systems in emotions. Philosophical Transactions of the Royal Society B-Biological Sciences 364: 2391–2404. 10.1098/rstb.2009.0058 PMC2865077 19620110

[27] Leslie KR, Johnson-Frey SH, Grafton ST (2004) Functional imaging of face and hand imitation: Towards a motor theory of empathy. Neuroimage 21: 601–607. 14980562 10.1016/j.neuroimage.2003.09.038

[28] Preston SD, de Waal FBM (2002) Empathy: Its ultimate and proximate bases. Behavioral and Brain Sciences 25: 1–58. 12625087 10.1017/s0140525x02000018

[29] Molnar-Szakacs I (2011) From actions to empathy and morality–A neural perspective. Journal of Economic Behavior & Organization 77: 76–85.

[30] Mascaro O, Csibra G (2012) Representation of stable social dominance relations by human infants. Proceedings of the National Academy of Sciences of the United States of America 109: 6862–6867. 22509020 10.1073/pnas.1113194109 PMC3344985

[31] Thomsen L, Frankenhuis WE, Ingold-Smith M, Carey S (2011) Big and mighty: Preverbal infants mentally represent social dominance. Science 331: 477–480. 21273490 10.1126/science.1199198 PMC3860821

[32] Kuhlmeier V, Wynn K, Bloom P (2003) Attribution of dispositional states by 12-month-olds. Psychological Science 14: 402–408. 12930468 10.1111/1467-9280.01454

[33] Hamlin JK, Wynn K, Bloom P (2007) Social evaluation by preverbal infants. Nature 450: 557–559. 18033298 10.1038/nature06288

[34] Hamlin JK, Wynn K (2011) Young infants prefer prosocial to antisocial others. Cognitive Development 26: 30–39. 21499550 10.1016/j.cogdev.2010.09.001 PMC3076932

[35] Fawcett C, Liszkowski U (2012) Infants anticipate others’ social preferences. Infant and Child Development 21: 239–249.

[36] Scarf D, Imuta K, Colombo M, Hayne H (2012) Social evaluation or simple association? Simple associations may explain moral reasoning in infants. Plos One 7: e42698. 22905161 10.1371/journal.pone.0042698 PMC3414446

[37] Hamlin JK, Wynn K, Bloom P (2010) Three-month-olds show a negativity bias in their social evaluations. Developmental Science 13: 923–929. 20977563 10.1111/j.1467-7687.2010.00951.x PMC2966030

[38] Nyström P (2008) The infant mirror neuron system studied with high density EEG. Social Neuroscience 3: 334–347. 18979389 10.1080/17470910701563665

[39] Aarts H, Gollwitzer PM, Hassin RR (2004) Goal contagion: Perceiving is for pursuing. Journal of Personality and Social Psychology 87: 23–37. 15250790 10.1037/0022-3514.87.1.23

[40] Spelke ES, Breinlinger K, Macomber J, Jacobson K (1992) Origins of knowledge. Psychological Review 99: 605–632. 1454901 10.1037/0033-295x.99.4.605

[41] Biro S, Csibra G, Gergely G (2007) The role of behavioral cues in understanding goal-directed actions in infancy. In: von Hofsten C, Rosander K, editors. From Action to Cognition. 303–322. 10.1016/S0079-6123(07)64017-5 17920439

[42] Good P (2005) Permutation, parametric, and bootstrap tests of hypotheses. New York: Springer.

[43] Warneken F, Tomasello M (2006) Altruistic helping in human infants and young chimpanzees. Science 311: 1301–1303. 16513986 10.1126/science.1121448

[44] Van Overwalle F, Baetens K (2009) Understanding others’ actions and goals by mirror and mentalizing systems: A meta-analysis. Neuroimage 48: 564–584. 19524046 10.1016/j.neuroimage.2009.06.009

[45] Gredebäck G, Melinder A (2010) Infants’ understanding of everyday social interactions: A dual process account. Cognition 114: 197–206. 19800056 10.1016/j.cognition.2009.09.004

[46] Brass M, Schmitt RM, Spengler S, Gergely G (2007) Investigating action understanding: Inferential processes versus action simulation. Current Biology 17: 2117–2121. 18083518 10.1016/j.cub.2007.11.057

[47] Gergely G, Csibra G (2003) Teleological reasoning in infancy: The naive theory of rational action. Trends in Cognitive Sciences 7: 287–292. 12860186 10.1016/s1364-6613(03)00128-1

[48] Sartori L, Begliomini C, Castiello U (2013) Motor resonance in left- and right-handers: evidence for effector-independent motor representations. Frontiers in Human Neuroscience 7: 33. 23408666 10.3389/fnhum.2013.00033 PMC3570897

[49] Csibra G (2008) Action mirroring and action interpretation: An alternative account. In: Haggard P, Rosetti Y, Kawato M, editors. Sensorimotor Foundations of Higher Cognition Attention and Performance XXII. Oxford: Oxford University Press. 435–459.

[50] Liepelt R, Cramon DYV, Brass M (2008) What is matched in direct matching? Intention attribution modulates motor priming. Journal of Experimental Psychology: Human Perception and Performance 34: 578–591. 18505325 10.1037/0096-1523.34.3.578

[51] Southgate V, Begus K (2013) Motor activation during the prediction of nonexecutable actions in infants. Psychological Science. In press. 10.1177/0956797612459766 PMC3938142 23678509

[52] Decety J (2010) To what extent is the experience of empathy mediated by shared neural circuits? Emotion Review 2: 204–207.

[53] Decety J, Michalska KJ, Kinzler KD (2012) The contribution of emotion and cognition to moral sensitivity: A neurodevelopmental study. Cerebral Cortex 22: 209–220. 21616985 10.1093/cercor/bhr111

[54] Fan Y, Han S (2008) Temporal dynamic of neural mechanisms involved in empathy for pain: An event-related brain potential study. Neuropsychologia 46: 160–173. 17825852 10.1016/j.neuropsychologia.2007.07.023

[55] Jackson PL, Decety J (2004) Motor cognition: A new paradigm to study self-other interactions. Current Opinion in Neurobiology 14: 259–263. 15082334 10.1016/j.conb.2004.01.020

[56] Masten CL, Morelli SA, Eisenberger NI (2011) An fMRI investigation of empathy for ‘social pain’ and subsequent prosocial behavior. Neuroimage 55: 381–388. 21122817 10.1016/j.neuroimage.2010.11.060

[57] Heberlein AS, Adolphs R (2004) Impaired spontaneous anthropomorphizing despite intact perception and social knowledge. Proceedings of the National Academy of Sciences of the United States of America 101: 7487–7491. 15123799 10.1073/pnas.0308220101 PMC409945

[58] Chartrand TL, Bargh JA (1999) The Chameleon effect: The perception-behavior link and social interaction. Journal of Personality and Social Psychology 76: 893–910. 10402679 10.1037//0022-3514.76.6.893

[59] Over H, Carpenter M (2009) Eighteen-month-old infants show increased helping following priming with affiliation. Psychological Science 20: 1189–1193. 19674388 10.1111/j.1467-9280.2009.02419.x

[60] Meltzoff AN (1995) Understanding the intentions of others - reenactment of intended acts by 18-month-old children. Developmental Psychology 31: 838–850. 25147406 10.1037/0012-1649.31.5.838 PMC4137788

[61] Kärtner J, Keller H, Chaudhary N (2010) Cognitive and social influences on early prosocial behavior in two sociocultural contexts. Developmental Psychology 46: 905–914. 20604610 10.1037/a0019718

[62] Meltzoff AN (2007) ‘Like me’: a foundation for social cognition. Developmental Science 10: 126–134. 17181710 10.1111/j.1467-7687.2007.00574.x PMC1852489

