# Correction: Infants Help a Non-Human Agent

**DOI:** 10.1371/annotation/138bc1aa-8891-4076-90bd-52b52ac9eab7

**Published:** 2013-11-07

**Authors:** Ben Kenward, Gustaf Gredebäck

Figures 1 and 2 were incorrectly switched. The image currently appearing as Figure 1 belongs with the title and legend for Figure 2, and the image currently appearing as Figure 2 belongs with the title and legend for Figure 1. The titles and legends are in correct order. 

